# Real Versus Sham-Based Neurodynamic Techniques in the Treatment of Cubital Tunnel Syndrome: A Randomized Placebo-Controlled Trial

**DOI:** 10.3390/jcm14062096

**Published:** 2025-03-19

**Authors:** Tomasz Wolny, Michał Wieczorek

**Affiliations:** 1 Musculoskeletal Elastography and Ultrasonography Laboratory, Institute of Physiotherapy and Health Sciences, The Jerzy Kukuczka Academy of Physical Education, Mikołowska 72A, 40-065 Katowice, Poland; 2 Department of Theoretical and Practical Basics of Physical Therapy, The Jerzy Kukuczka Academy of Physical Education, Mikołowska 72A, 40-065 Katowice, Poland; m.wieczorek@awf.katowice.pl

**Keywords:** cubital tunnel syndrome, physical therapy modalities, manual therapy, sham treatment, physiotherapy techniques

## Abstract

**Background/Objective:** To assess the effectiveness of therapy based on sliding and tensioning neurodynamic techniques in the conservative treatment of mild and moderate forms of cubital tunnel syndrome (CuTS) compared to sham therapy. **Methods:** A single-blinded, randomized placebo-controlled trial. The study was conducted at several medical clinics. Individuals diagnosed with CuTS (initially 136 subjects, of whom 91 completed the full protocol) participated in the experiment. In the experimental group (MT), sliding and tensioning neurodynamic techniques were applied, whereas in the control group (ST), a sham therapy was used, involving the performance of neurodynamic techniques in an intermediate position without following the specific neurodynamic sequence for the ulnar nerve. The therapy was administered five times per week over the course of 10 sessions. All participants underwent assessments, including nerve conduction studies, ultrasound imaging (cross-sectional area and shear modulus), pain levels, two-point discrimination sensation, cutaneous sensory perception threshold, symptoms, ability to perform certain activities, and changes in improvement following treatment. **Results:** A baseline assessment revealed no significant inter-group differences in all examined parameters (*p* > 0.05). After therapy, there was a statistically significant intra-group improvement in all parameters tested (*p* < 0.01). In the MT group, the intra-group differences were significant across all parameters tested (*p* < 0.01). However, in the ST group (sham therapy), only the shear modulus showed statistically significant changes, while the other tested parameters remained unchanged. **Conclusions:** Neurodynamic techniques demonstrate superior therapeutic effects compared to sham therapy in the treatment of mild to moderate forms of CuTS.

## 1. Introduction

Cubital tunnel syndrome (CuTS) is the second most common peripheral neuropathy of the upper limb with a prevalence ranging from 2% to 6% [1,2]. Initial symptoms include paresthesias and mild hypoesthesia of the fifth and fourth fingers occurring paroxysmally and related to the position of the elbow [3]. Over time, motor dysfunction develops, manifesting as weakness and atrophy of the intrinsic muscles of the hand [4]. This progression can significantly impair hand function related to both activities of daily living and work, leading to progressive disability and reducing health-related quality of life [5]. Given its high prevalence, CuTS is a major medical, economic, and social challenge.

Peripheral neuropathies, often referred to as ‘entrapment syndrome’, indicates nerve compression by the surrounding tissues [6]. At the elbow, the sensitive anatomical position of the ulnar nerve induces significant tension and increased compression of the nerve [7]. Under normal conditions, the nervous system has adaptive mechanisms that allow it to function efficiently [6,7]. Anatomical changes due to trauma, degenerative changes, and occupational risk factors can compromise these adaptive mechanisms, leading to the onset of CuTS symptoms. Repeated elbow flexion and chronic external pressure on the superficially lying ulnar nerve result in nerve entrapment and impaired mobility and are considered to be the pathogenesis of CuTS [8]. Chronic nerve compression can lead to circulatory disorders, epineural and endoneural edema, and nerve fibrosis [9]. Therefore, improving nerve mechanics seems to be one of the key elements in the treatment of this peripheral neuropathy.

CuTS is difficult to diagnose due to the large spectrum of clinical symptoms [10]. Some researchers have advised about the lack of a gold standard in the diagnosis of CuTS [11]. In clinical practice, diagnosis is based on a combined analysis of the patient’s history, clinical symptoms, physical examination, electrophysiological testing and ultrasound imaging (US) [12]. US is a promising tool in the diagnosis of peripheral neuropathies due to the large spectrum of clinical symptoms and the limitations of electrophysiological testing [13,14]. It should be emphasized that there is no widely accepted ultrasound-defined criteria for the diagnosis of CuTS. The work by Tomažin et al. (2024) provides valuable insights into ultrasound’s role beyond cross-sectional area assessment, particularly in carpal tunnel syndrome, which could offer a comparative perspective on imaging modalities in CuTS [14]. Thus, conducting further research on the role of US in the diagnosis of CuTS seems highly desirable.

The management of CuTS includes both conservative and surgical approaches [15]. Conservative treatment is usually recommended as first-line therapy [4]. This treatment includes the modification of activities, orthoses, non-steroidal drugs, local steroid injection, and physiotherapy approaches [5,16,17,18]. However, as noted by Mezian et al. [12], the conservative treatment of CuTS relies more on the therapist’s empirical experience than high-quality scientific evidence. Surgical treatment is usually reserved for severe cases of CuTS [19]. Palmer and Hughes [16] highlight that no ‘gold standard’ of surgical treatment has been developed so far. As emphasized by Caliandro et al. [20], there are currently no guidelines to determine the appropriate intervention.

Physiotherapy appears to be one of the first forms of conservative treatment for CuTS. To date, one systematic review and one critical review of the literature have shown the effectiveness of physiotherapy in the treatment of CuTS [5,18]. The authors emphasize that there are a small number of randomized clinical trials evaluating the effectiveness of physiotherapy in the treatment of CuTS, and it is difficult to conclusively determine the efficacy of specific interventions. Three randomized clinical trials [19,21,22] and three case studies [23,24,25] evaluated the effectiveness of neurodynamic techniques. While these studies reported beneficial therapeutic effects, neurodynamic techniques were only one component of the therapeutic programs. This does not clearly indicate that neurodynamic techniques are effective in CuTS. It is therefore difficult to assess the real contribution of neurodynamic techniques to the improvement of CuTS patients. Given the potential benefits of neurodynamic techniques in the treatment of other peripheral neuropathies [26,27], we decided to evaluate the efficacy of these techniques in the treatment of CuTS. Therefore, the aim of this study is to evaluate the effectiveness of neurodynamic techniques compared to sham therapy in the physiotherapy management of CuTS.

## 2. Materials and Methods

### 2.1. Ethics

The Bioethics Committee for Scientific Studies at the Academy of Physical Education in Katowice authorized the study on 14 November 2019 (Decision No. 8/2019). All study procedures were performed in accordance with the Declaration of Helsinki. The clinical trial was registered at the Australian New Zealand Clinical Trials Registry (ANZCTR) under number ACTRN12621001623866. All patients were informed about the study objectives and procedures and about their right to withdraw at any time without providing a reason. Written informed consent was obtained from all participants.

### 2.2. Study Design

The study was designed as a single-blinded, randomized placebo-controlled trial and conducted between 2022 and 2023. Each patient received a diagnosis of CuTS based on medical history, clinical examination, and electrophysiological diagnosis made by a physician, and they also met the inclusion criteria, which qualified them to participate in the experiment. Only patients with mild and moderate CuTS (based on clinical symptoms according to McGowan’s classification) were eligible for the study [28]. Participants who qualified for the study were randomly divided into one of two groups: neurodynamic techniques (NT) or sham therapy (ST).

### 2.3. Sample Size

The necessary sample size was calculated based on preliminary results from 15 participants. The pain scale data along with our previous calculations were used to determine the sample size [27]. The calculation of sample size was based on an alpha of 0.05 and a statistical power of 0.8 [29]. Based on this analysis, we aimed to recruit about 82 participants (41 for each treatment group).

### 2.4. Participants

**Figure 1:**
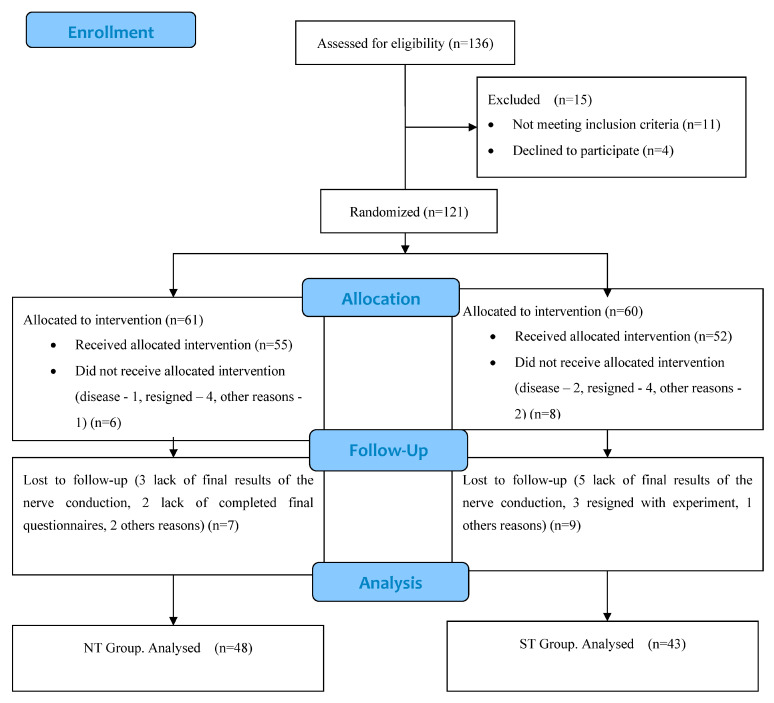
Flow diagram of phases through clinical trial.

Initially, 136 participants with CuTS were considered for inclusion in the study. Of these, 15 were excluded because they did not meet the inclusion criteria or declined to participate. In the next stages of the experiment, another 16 individuals were excluded. Ultimately, 91 participants completed the entire protocol (Figure 1).

### 2.5. Protocol

#### 2.5.1. Diagnostic Criteria of CuTS

All participants were diagnosed with CuTS by a few physicians. The standardization of diagnosis was based on the following criteria.

The inclusion criteria are presented below:

(a) Clinical symptoms of peripheral neuropathy of the ulnar nerve (pain, numbness, and tingling),
(b) Objective clinical symptoms of peripheral neuropathy of the ulnar nerve (positive elbow flexion test; Tinel’s sign; one or two points in the McGowan classification),
(c) Disturbances in the ulnar nerve sensation tests (two-point discrimination sense, sensory threshold test),
(d) Changes in ultrasound measurement (increased cross-sectional area, greater stiffness of the ulnar nerve),
(e) Below-normal results of the nerve conduction study (motor conduction velocity < 49.3 m/s).

CuTS was diagnosed when at least two symptoms and nerve conduction abnormalities were present.

The main exclusion criteria for the study were no symptoms of cubital tunnel syndrome, previous surgical treatment, mental illness, cervical radiculopathy, rheumatoid disease, diabetes, pregnancy, contraindications to therapy, and lack of cooperation or consent to participate in the study.

#### 2.5.2. Randomization and Allocation

Each patient who met the inclusion criteria was enrolled in the study and randomly assigned to one of two parallel groups (NT and ST) using a computer random number generator. Cards labeled with the numbers “1” and “2” were generated and placed in an envelope. Participants who drew cards marked “1” were allocated to the NT group and those who drew “2” were assigned to the ST group. The assistants who performed the randomization were not part of the research team and were not involved in the experiment.

#### 2.5.3. Blinding Procedures

The diagnosis of CuTS was confirmed by a physician before participants were allocated to their respective groups. The diagnosing physician was not a member of the research group. Nerve conduction studies were performed in an independent laboratory as part of standard procedures, and laboratory staff were not informed about the conducted experiment. The remaining examinations were performed by physiotherapists blinded to the allocation of patients to the research groups. The physiotherapists administering neurodynamic techniques were experienced in these methods, while those conducting sham therapy underwent prior training to ensure the procedures were performed without activating the ulnar nerve. All investigators had more than 10 years of experience as physiotherapists, including experience in working with individuals with peripheral neuropathies. After two weeks of therapy, the participants were re-examined by the same physiotherapists who had conducted the baseline assessment. The final NCS studies were performed in the same laboratory.

### 2.6. Outcome Measures

#### 2.6.1. Primary Outcomes

Nerve conduction studies of the ulnar nerve were conducted in an electroneurography laboratory by an experienced neurophysiologist as ordered by the physician. The temperature of the testing room was maintained between 24 °C and 26 °C. Before the examination, patients were acclimated for 10 to 15 min. The NCS study was performed using superficial electrodes. The following values for the ulnar nerve were accepted as normative, as recommended by the laboratory (and with cut-off points of EMG national guidelines): motor conduction velocity ≥ 49.3 m/s, sensory conduction velocity ≥ 54.9 m/s, and distal motor latency ≤ 3.4 milliseconds. NCS was performed at baseline (1–2 weeks) and after treatment (1–2 weeks).

Ultrasound imaging measurements were performed with a Hologic Supersonic Mach 30 ultrasound scanner (Supersonic Imagine, Aix En Provence, France) using a linear transducer array (5–18 MHz; Super Linear SL18-5, Supersonic Imagine). All ultrasound examinations and measurements (patient positioning, measurement sites, transducer positioning, measurement methodology, etc.) followed the approach described by Wolny et al. [30]. The cross-sectional area (CSA) and shear modulus were assessed in four positions of the elbow joint (full extension, 45° flexion, 90° flexion, and maximum flexion). All measurements were taken in the cubital tunnel. CSA was recorded in square millimeters (mm^2^) and shear modulus was recorded in kilopascals (kPa). The average values of three measurements were used for analysis. Ultrasound assessments were performed both at baseline and after treatment.

A numerical pain rating scale (NPRS) was used to assess pain levels (0 = no pain, 10 = maximum pain) [31]. Participants were asked to rate their worst pain level during the day and at night over the past week. In cases of bilateral CuTS, pain was assessed separately for each limb. Pain assessment was performed both at baseline and after treatment.

#### 2.6.2. Secondary Outcomes

The examination of static two-point discrimination sensation (2PD) was performed using a standardized Dellon discriminator (Baseline, 2-Point DiscrIm-A-Gon^TM^, White Plains, NY, USA). The device comprises two plastic discs, each containing a series of metal pins separated by distances ranging from 1 to 15 mm. The discriminator spikes were applied at previously marked locations according to the methodology proposed by Wolny et al. [32]. The 2PD sensation test was performed on the fingertips of the fourth and fifth fingers. The discriminator was applied to the skin without additional pressure, and the shortest distance between discriminator spikes for which the subject provided a “two” response in three consecutive measurements was recorded in millimeters and used in the main analysis. Static 2PD was performed both at baseline and after treatment. Previous studies have shown a high reliability of the 2PD test [33].

A TOUCH TEST device (North Coast Medical, Inc., Morgan Hill, CA, USA) was used to evaluate the cutaneous sensory perception threshold (CSPT). The device consists of five monofilaments calibrated to produce a specific force in grams: green (size 2.83), 0.07 g; blue (size 3.61), 0.4 g; purple (size 4.31), 2.0 g; pink (size 4.56), 4.0 g; and red (size 6.65), 300 g. The following scale was used to analyze the CSPT: 0—no sensation during stimulation; 1—0.07 g monofilament; 2—0.4 g monofilament; 3—2.0 g monofilament; 4—4.0 g monofilament; and 5—300 g monofilament [33]. The monofilaments were applied at previously marked locations according to the methodology proposed by Wolny et al. [32]. The test was performed on the fourth and fifth fingers. The thinnest monofilament of three measurements that the subject indicated during stimulation was recorded, and this value was used in the analysis. CSPT was performed at baseline and after treatment. The high reliability of the CSPT test has been demonstrated in previous studies [32].

The Quick Disabilities of Arm Shoulder and Hand (QDASH) questionnaire was used to assess symptoms and ability to perform activities. This self-assessment questionnaire consists of 11 questions assessing the ability to perform activities, the impact on normal social activities, limitations in daily activities, and severity of symptoms during the day and at night. Scores range from 1 for no problems to 5 for maximum problems [34]. The higher scores indicate worse conditions. Participants completed QDASH at baseline and after treatment.

The Self-Complete of Leeds Assessment of Neuropathic Symptoms and Signs (S-LANSS) for the Polish population was used to assess neuropathic pain [35]. The questionnaire consists of seven items with a maximum score of 24 points (higher scores indicating worse conditions). A score of 12 points or more indicates that the pain is predominantly neuropathic in origin. Participants completed the S-LANSS questionnaire at baseline and after treatment.

The GROC is a self-reported outcome measure used to gauge the patient’s perceived improvement following treatment. It is a 15-point scale ranging from −7 to +7 (a very great deal worse to a very great deal better) with a minimum clinically important difference set at 3 points [36]. The GROC was assessed after treatment.

### 2.7. Intervention

During the first therapeutic session, the physiotherapist provided one-time instruction on how to avoid activities that provoke CuTS symptoms, especially prolonged flexed elbow, leaning on the elbow for a long time, and keeping the elbow straight while sleeping in both groups (NT and ST).

In the NT group, manual therapy was based on sliding and tensioning neurodynamic techniques of the ulnar nerve. Neurodynamic techniques were performed in two positions and neurodynamic sequences for the ulnar nerve:

- Neurodynamic techniques for ulnar nerve 1 (NTUN1)—position: supine; neurodynamic sequence: wrist and finger extension, forearm pronation, shoulder external rotation, elbow flexion, shoulder girdle depression, shoulder abduction; neurodynamic techniques: one-direction distal sliding mobilization (movement—rhythmic hand flexion and extension—large amplitude of motion), one-direction distal tensioning mobilization (movement—rhythmic hand flexion and extension—small amplitude of motion at the end of the movement), one-direction proximal sliding mobilization (movement—rhythmic elbow flexion and extension—large amplitude of motion), one-direction proximal tensioning mobilization (movement—rhythmic elbow flexion and extension—small amplitude of motion at the end of the movement).
- Neurodynamic techniques for ulnar nerve 2 (NTUN2)—position: supine; neurodynamic sequence: wrist and finger extension and radial adduction, forearm pronation, shoulder internal rotation, elbow extension, shoulder girdle depression, shoulder abduction; neurodynamic techniques: one-direction distal sliding mobilization (movement—rhythmic hand flexion and extension—large amplitude of motion), one-direction distal tensioning mobilization (movement—rhythmic hand flexion and extension—small amplitude of motion at the end of the movement), one-direction proximal sliding mobilization (movement—rhythmic elbow flexion and extension—large amplitude of motion), one-direction proximal tensioning mobilization (movement—rhythmic elbow flexion and extension—small amplitude of motion and the end of the movement).

The standard protocol included one series of 60 repetitions of sliding and tensioning distal and proximal neurodynamic techniques in both positions (NTUN1, NTUN2) separated by inter-series intervals of 15 s five times a week for 10 sessions. The approximate duration of each session was around 30 min.

In the ST group, sham manual therapy was based on sham sliding and the sham tensioning neurodynamic techniques of the ulnar nerve. Sham neurodynamic techniques (placebo therapy) were performed in two positions without using neurodynamic sequences for the ulnar nerve:

(a) Sham neurodynamic techniques for ulnar nerve 1 (sNTUN1)—position: supine; no neurodynamic sequence: wrist and finger in the neutral position, forearm pronation, shoulder external rotation, elbow flexion, shoulder girdle neutral position, no shoulder abduction; sham neurodynamic techniques: sham one-direction distal sliding mobilization (movement—rhythmic hand flexion and extension—large amplitude of motion), sham one-direction distal tensioning mobilization (movement—rhythmic hand flexion and extension—small amplitude of motion at the end of the movement), sham one-direction proximal sliding mobilization (movement—rhythmic elbow flexion and extension—large amplitude of motion), sham one-direction proximal tensioning mobilization (movement—rhythmic elbow flexion and extension—small amplitude of motion at the end of the movement).
(b) Sham neurodynamic techniques for ulnar nerve 2 (sNTUN2)—position: supine; no neurodynamic sequence: wrist and finger in neutral position, forearm pronation, shoulder in neutral position, elbow extension, shoulder girdle in neutral position, no shoulder abduction; sham neurodynamic techniques: sham one-direction distal sliding mobilization (movement—rhythmic hand flexion and extension—large amplitude of motion), sham one-direction distal tensioning mobilization (movement—rhythmic hand flexion and extension—small amplitude of motion at the end of the movement), sham one-direction proximal sliding mobilization (movement—rhythmic elbow flexion and extension—large amplitude of motion), sham one-direction proximal tensioning mobilization (movement—rhythmic elbow flexion and extension—small amplitude of motion at the end of the movement).

The standard protocol included one series of 60 repetitions of sliding and tensioning distal and proximal neurodynamic techniques in both positions (sNTUN1, sNTUN2) separated by inter-series intervals of 15 s five times a week for 10 sessions. The approximate duration of each session was around 30 min.

It should be emphasized that all participants with CuTS who received sham therapy received standard treatment immediately after the experiment was completed.

### 2.8. Statistical Analysis

Data were analyzed using the Statistica 13.1 software package. The basic parameters were compared between groups using the independent t-test (age, body mass, height, and body mass index in kilograms per square meter) and the Chi-square test (gender distribution, side of hand dominance, side of asymptomatic and symptomatic hand, and the number of affected cubital tunnel syndrome hands—one hand or both hands). A one-way analysis of variance (ANOVA) for repeated measurements was used to evaluate the main effects of nerve conduction study, CSA, shear modulus, pain, 2PD, CSPT, Q-DASH, and S-LANSS between groups. For between-group differences, Tukey’s post hoc test was used. Significant results are reported as mean differences with a 95% confidence interval (CI). For all analyses, the threshold of the *p*-value considered significant was set at <0.05.

## 3. Results

### 3.1. Participant Characteristics at Baseline

The final analysis included 91 participants (48 in NT and 43 in SM group). At baseline, the groups were similar in terms of sex, age, body mass, body height, BMI, unilateral/bilateral CuTS, symptomatic/asymptomatic hand, dominant hand, and symptom duration. Detailed data are shown in Table 1. 

### 3.2. Inter- and Intra-Group Comparison of Clinical Symptoms

ANOVA performed on McGowan classification, elbow flexion test, and Tinel’s sign showed significant between-group differences (*p* < 0.01), a significant treatment effect (*p* < 0.01), and significant between-group interactions (*p* < 0.01). The results of Tukey post hoc tests are shown in Table 2.

### 3.3. Inter- and Intra-Group Comparison of Primary Outcomes

ANOVA performed on motor conduction velocity of the ulnar nerve revealed a group difference, an effect of therapy, and an interaction between group and therapy (*p* < 0.01 in all cases).

In the assessment of the cross-sectional area at elbow extension, ANOVA showed no significant between-group differences (*p* > 0.05), a significant treatment effect (*p* < 0.01), and significant between-group interactions (*p* < 0.01). Similar effects were noted at 90° and full flexion of the elbow. ANOVA performed on the cross-sectional area at 45° flexion of the elbow showed significant between-group differences (*p* < 0.01), a significant treatment effect (*p* < 0.01), and significant between-group interactions (*p* < 0.01).

In the assessment of shear modulus at elbow extension, and 45°, 90°, and full flexion, ANOVA showed significant between-group differences (*p* < 0.01), a significant treatment effect (*p* < 0.01), and significant between-group interactions (*p* < 0.01).

ANOVA performed on diurnal and nocturnal pain showed significant between-group differences (*p* < 0.01), a significant treatment effect (*p* < 0.01), and significant between-group interactions (*p* < 0.01) (Table 3).

ANOVA performed on 2PD sensation and CSPT of the fourth and fifth finger showed significant between-group differences (*p* < 0.01), a significant treatment effect (*p* < 0.01), and significant between-group interactions (*p* < 0.01). ANOVA performed on the Q-DASH questionnaire and S-LANSS scale showed significant between-group differences (*p* < 0.01), a significant treatment effect (*p* < 0.01), and significant between-group interactions (*p* < 0.01) (Table 4).

## 4. Discussion

The aim of this study was to assess the effectiveness of neurodynamic techniques compared to sham therapy in the treatment of CuTS. The findings demonstrated that neurodynamic techniques have beneficial therapeutic effects, as evidenced by improvements in objective measurements such as nerve conduction, ultrasound measurements, provocation tests, discriminative sensation and sensory threshold. Furthermore, the subjective assessment of individuals with CuTS, including the degree of upper limb disability and neuropathic pain, indicates a beneficial effect of neurodynamic techniques compared to sham therapy.

Studies conducted so far have not clearly demonstrated the beneficial effects of neurodynamic techniques in the treatment of CuTS [5,14]. This is because neurodynamic techniques were most often used as part of a therapeutic program rather than as a stand-alone therapeutic tool. This is also highlighted by the authors of studies who emphasized in the limitations that it is difficult to determine whether neurodynamic techniques provide the greatest therapeutic benefit if other therapeutic tools were used [23].

Only one study used neurodynamic techniques as a stand-alone therapeutic tool [19]. Although there was a significant improvement, the same effect was observed in the other groups. It is important to highlight that such written information was provided to all the groups surveyed, so it is difficult to determine what actually contributed to the improvement in the final survey. The authors emphasize that based on recommendations from the literature, they anticipated a positive effect from neurodynamic techniques and were surprised to find no additional benefits [19].

Furthermore, it is worth emphasizing that only three studies using neurodynamic techniques have been conducted as randomized clinical trials [19,21,22]. In a study by Sverlov et al. [19], neurodynamic techniques were performed as a home therapy program. There is always a concern with home exercises because it may not be performed correctly, and sometimes the adherence to the prescribed therapy may be inconsistent or incomplete. In a study by Garber et al. [21], neurodynamic techniques were combined with an orthosis and compared to ultrasound therapy. Only muscle strength was measured. After therapy, significant increases were obtained without group differences [21]. Unfortunately, focusing solely on muscle strength does not show how other important symptoms of CuTS have changed. Galal et al. [22]. combined neurodynamic techniques, ultrasound therapy, strength exercises and dry cupping therapy. The researchers demonstrated a significant reduction in pain, increased strength, and improved function. Unfortunately, it is difficult to assess the effectiveness of the neurodynamic techniques, as the therapeutic program consisted of several tools [21]. It should be emphasized that no definite conclusions can be drawn from any of the above randomized clinical trials regarding the effectiveness of neurodynamic techniques in the conservative treatment of CuTS.

Additionally, three case studies evaluated the use of neurodynamic techniques in the treatment of CuTS [19,20,21]. Oskay et al. [23] evaluated the effectiveness of a therapeutic program consisting of cold, neurodynamic techniques and exercises in seven individuals with CuTS. The authors concluded that conservative treatment may be beneficial for selected individuals with mild to moderate CuTS. They also emphasize that therapy incorporating sliding and tensioning neurodynamic techniques improves nerve slide and nerve tissue mobility. The authors emphasized that the therapy for individuals with CuTS should aim to prevent symptom-provoking positions and improve the nerve biomechanics and function of the entire upper limb, which underscores the importance of using a comprehensive therapeutic approach. It is important in clinical trials to affect not only the symptoms but also the underlying causes of the condition in a comprehensive way. Unfortunately, in clinical trials, it is difficult to assess which of the therapeutic tools used were effective and to what extent they improved the patient’s condition. Fernández-de-Las-Peñas et al. [24] evaluated the effect of percutaneous electrical stimulation on the condition of a female patient with CuTS. The neurodynamic techniques were included only as an additional recommendation for home practice. Unfortunately, in both works, we do not know which therapeutic tools improve the patient’s condition. Coppieters et al. [25]. reported significant improvement in a 17-year-old woman with acute CuTS using neurodynamic techniques, segmental spinal manipulation, and exercises. Following therapy, the patient experienced a reduction in pain and improved limb function. Unfortunately, the patient’s assessment was limited to a functional examination. No nerve conduction study or ultrasound examination was performed. It is therefore difficult to confirm a certain diagnosis of CuTS. Nevertheless, it is important and noteworthy that therapy based on neurodynamic techniques had a beneficial therapeutic effect.

To our knowledge, this study is the first to evaluate the effectiveness of neurodynamic techniques used as the only therapeutic tool in the treatment of mild to moderate forms of CuTS. The therapeutic effects obtained indicate that the use of neurodynamic techniques has a beneficial effect in the treatment of subjects with CuTS. A combination of sliding and tensioning techniques was introduced to improve nerve sliding in relation to surrounding tissues, reduce nerve swelling but also improve slow and fast axonal transport. As emphasized by Coppieters et al. [25], the aim of therapy in CuTS is to normalize the sensitivity of the nervous system and restore normal nerve biomechanics. Neurodynamic techniques reduce intra- and extra-neural swelling, improve blood circulation, and restore the mobility and elasticity of the nerve tissue. Neurodynamic techniques have also been indicated to enhance axoplasmic flow, increase motor unit recruitment, improve muscle strength, and reduce pain intensity [37,38]. Studies indicate that in the early phase of nerve damage, inflammation and edema restrict nerve sliding, increase intraneural pressure, and obstruct blood flow and axonal transport, causing pain and paresthesias [39,40]. Furthermore, intraneural and perineural fibrosis also decreases nerve tissue elasticity and extensibility [41]. Therefore, improving circulation, axonal transport, and nerve mobility are essential for the functional and structural integrity of neurons, which can be achieved through both sliding and tensioning neurodynamic techniques.

It is worth emphasizing that proper diagnosis is important in the case of CuTS. Anatomical differences, different places of ulnar nerve compression, a wide spectrum of symptoms, and the need to differentiate this problem from other neuropathies of the ulnar nerve often pose many problems regarding diagnosis. Also, the assessment of nerve conduction in the case of CuTS is not the gold standard [12,42]. Researchers point to low sensitivity, the risk of false-negative results, and the invasiveness of nerve conduction study. Therefore, it is necessary to emphasize the special role of ultrasound examination in the accurate diagnosis of CuTS [14,30].

We believe that novelty is the strength of our research. This is the first randomized clinical trial to evaluate the effectiveness of neurodynamic techniques in the treatment of CuTS. We have described in detail the therapy protocol using neurodynamic techniques, and it can be readily implemented in clinical practice by physiotherapists. The study included a relatively large group of individuals with CuTS. The findings demonstrate the positive impact of neurodynamic techniques in the conservative treatment of mild to moderate forms of CuTS.

In future studies, it would be worthwhile to evaluate the effectiveness of neurodynamic techniques compared to other therapeutic interventions. It would also be useful to compare the effectiveness of sliding and tensioning techniques applied separately to determine their therapeutic potential.

### Study Limitations

The main limitation of this study is the lack of a double-blind design. Individuals with CuTS were blinded to the therapy they received, but the physiotherapists were fully aware of the therapy they administered. Another limitation is the lack of assessment of the long-term effects of the therapy. Unfortunately, such a study would be unethical.

## 5. Conclusions

The use of neurodynamic techniques in the conservative treatment of individuals with CuTS is more effective than sham therapy. Significant improvements were obtained both in the objective parameters and in the subjective symptoms.

## Figures and Tables

**Table 1:** Between-group comparisons for participants characteristics at baseline.

Characteristics	NT Group (n = 48)	ST Group (n = 43)	*p*-Value *
Women/Men (n, %)	16 (33%)/32 (67%)	16 (37%)/27 (63%)	0.68 ^a^
Age (mean, SD, min–max), year	54.4; 6.97; 40–68	53.3; 6.52; 43–66	0.44 ^b^
Body mass (mean, SD, min–max), kg	77.1; 13.1; 49–102	72.6; 11.3; 51–94	0.18 ^b^
Body height (mean, SD, min–max), cm	174; 9.74; 157–188	173; 9.73; 148–187	0.64 ^b^
BMI (mean, SD, min–max), kg/m^2^	24.8; 2.66; 20.2–32.4	24.1; 2.25; 20.1–30.5	0.10 ^b^
CuTS unilateral/bilateral (n, %)	43 (90%)/5 (10%)	40 (93%)/3 (7%)	0.72 ^a^
Symptomatic hand right/left (n, %)	25 (47%)/28 (53%)	20 (43%)/26 (57%)	0.71 ^a^
Asymptomatic hand right/left (n, %)	23 (51%)/22 (49%)	23 (57%)/17 (43%)	0.63 ^a^
Dominant hand right/left (n, %)	41 (85%)/7 (16%)	38 (88%)/5 (12%)	0.67 ^a^
Symptoms duration (mean, SD, min–max), month	11.1; 2.54; 7–17	11.4; 2.68; 7–18	0.29 ^b^

NOTE. Values are mean ± SD (minimum-maximum), n (%), or as otherwise indicated. Abbreviations: NT—neurodynamic techniques; ST—sham therapy; BMI—body mass index; n—number of participants; %—percentage value; SD—standard deviation; ^a^ Chi-square test; ^b^ Student’s *t*-test; * Statistically significant difference.

**Table 2:** Inter and intra-group comparison—clinical symptoms.

Clinical Tests	Group	NT Group (n = 48)		ST Group (n = 43)		Inter-Groups Difference *p*; 95% CI
	Time Point	Mean (SD)	Intra-Group Differences *p*	Mean (SD)	Intra-Group Differences *p*	
McGowan classification (scale 1–3)	Baseline Final	1.51 (0.5) 0.49 (0.5)	B vs. F—0.0001 *	1.5 (0.5) 1.3 (0.59)	B vs. F—0.0087 *	0.99 −0.19 to 0.21 0.0000 * −1.03 to −0.59
Elbow flexion test (0–60 s)	Baseline Final	22.3 (5.96) 40.4 (11.7)	B vs. F—0.0001 *	21.9 (6.71) 22.6 (5.51)	B vs. F—0.88	0.99 −2.08 to 2.97 0.0000 * 13.9 to 21.4
Tinel’s sign (0—negative, 1—positive)	Baseline Final	0.81 (0.39) 0.16 (0.37)	B vs. F—0.0001 *	0.71 (0.45) 0.58 (0.49)	B vs. F—0.02	0.72 −0.07 to 0.26 0.0000 * −0.59 to −0.24

NOTE. Values are mean ± SD (range) or as otherwise indicated. Abbreviation: NT—neurodynamic techniques; ST—sham therapy; n—number of participants; *p*—significance level, SD—standard deviation, CI—confidence interval, B—baseline, F—final. * Statistically significant difference.

**Table 3:** Inter and intra-group comparison—primary outcomes.

Clinical Tests	Group	NT Group (n = 48)		ST Group (n = 43)		Inter-Groups Difference *p*; 95% CI
	Time Point	Mean (SD)	Intra-Group Differences *p*	Mean (SD)	Intra-Group Differences *p*	
Motor conduction velocity (m/s)	Baseline Final	35.1 (6.28) 45.6 (6.65)	B vs. F—0.0001 *	36.1 (4.38) 36.2 (4.47)	B vs. F—0.99	0.89 −3.01 to 1.36 0.0000 * 7.24 to 11.8
Ultrasound cross-sectional area at elbow extension (mm^2^)	Baseline Final	10.9 (1.52) 9.42 (1.65)	B vs. F—0.0001 *	10.7 (1.13) 10.6 (1.08)	B vs. F—0.80	0.88 −0.33 to 0.75 0.0002 * −1.84 to −0.71
Ultrasound cross-sectional area 45° flexion (mm^2^)	Baseline Final	10.8 (1.51) 9.19 (1.41)	B vs. F—0.0001 *	10.8 (1.08) 10.8 (1.21)	B vs. F—0.99	0.99 −0.55 to 0.51 0.0000 * −2.19 to −1.14
Ultrasound cross-sectional area 90° flexion (mm^2^)	Baseline Final	10.4 (1.56) 10.1 (1.46)	B vs. F—0.0001 *	10.3 (1.31) 10.3 (1.35)	B vs. F—0.98	0.98 −0.46 to 0.69 0.86 −0.79 to 0.33
Ultrasound cross-sectional area at full flexion (mm^2^)	Baseline Final	11.1 (1.41) 10.5 (1.24)	B vs. F—0.0001 *	10.9 (1.48) 10.8 (1.12)	B vs. F—0.15	0.99 −0.44 to 0.58 0.70 −0.75 to 0.19
Ultrasound shear modulus at elbow extension (kPa)	Baseline Final	29.8 (4.93) 21.4 (5.19)	B vs. F—0.0001 *	30.2 (4.25) 27.4 (4.08)	B vs. F—0.0002 *	0.96 −2.33 to1.36 0.0000 * −7.93 to −4.16
Ultrasound shear modulus 45° flexion (kPa)	Baseline Final	89.6 (22.4) 51.6 (15.9)	B vs. F—0.0001 *	88.9 (17.1) 82.8 (16.9)	B vs. F—0.0024 *	0.99 −7.39 to8.73 0.0001 * −37.7 to −24.6
Ultrasound shear modulus 90° flexion (kPa)	Baseline Final	111.8 (17.4) 88.3 (18.5)	B vs. F—0.0001 *	112.4 (12.6) 109.5 (12.2)	B vs. F—0.24	0.99 −6.73 to5.57 0.0000 * −27.5 to −14.8
Ultrasound shear modulus at full flexion (kPa)	Baseline Final	209.9 (83.9) 139.1 (43.6)	B vs. F—0.0001 *	204.6 (62.4) 201.5 (62.2)	B vs. F—0.93	0.97 −24.6 to35.1 0.0001 * −83.7 to −41.2
Diurnal pain NPRS (0—no pain; 10—maximum pain)	Baseline Final	4.77 (1.01) 1.09 (0.94)	B vs. F—0.0001 *	4.63 (0.95) 4.41 (0.83)	B vs. F—0.17	0.88 −0.25 to 0.53 0.0000 * −3.67 to −2.96
Nocturnal pain NPRS (0—no pain; 10—maximum pain)	Baseline Final	2.56 (0.61) 0.64 (0.68)	B vs. F—0.0001 *	2.84 (0.69) 2.71 (0.71)	B vs. F—0.40	0.18 −0.54 to −0.02 0.0000 * −2.35 to −1.79

NOTE. Values are mean ± SD (range) or as otherwise indicated. Abbreviation: NT—neurodynamic techniques; ST—sham therapy; NPRS—numerical pain rating scale; m/s—meter per second; mm^2^—square millimeters; kPa—kilopascals; n—number of participants; *p*—significance level, SD—standard deviation, CI—confidence interval, B—baseline, F—final. * Statistically significant difference.

**Table 4:** Inter and intra-group comparison—secondary outcomes.

Clinical Tests	Group	NT Group (n = 48)		ST Group (n = 43)		Inter-Groups Difference *p*; 95% CI
	Time Point	Mean (SD)	Intra-Group Differences *p*	Mean (SD)	Intra-Group Differences *p*	
2PD—4th finger (mm)	Baseline Final	6.83 (0.97) 5.41 (1.26)	B vs. F—0.0001 *	6.76 (1.13) 6.73 (1.11)	B vs. F—0.99	0.99 −0.35 to 0.48 0.0000 * −1.81 to −0.84
2PD—5th finger (mm)	Baseline Final	7.05 (0.63) 5.81 (0.72)	B vs. F—0.0001 *	7.31 (0.61) 7.16 (0.57)	B vs. F—0.56	0.24 −0.49 to 0.01 0.0000 * −1.62 to −1.09
CSPT—4th finger (scale 0–4)	Baseline Final	3.04 (0.66) 1.54 (0.57)	B vs. F—0.0001 *	2.91 (0.71) 2.85 (0.66)	B vs. F—0.93	0.74 −0.13 to 0.41 0.0000 * −1.56 to −1.06
CSPT—5th finger (scale 0–4)	Baseline Final	3.06 (0.49) 1.74 (0.62)	B vs. F—0.0001 *	3.04 (0.58) 3.01 (0.57)	B vs. F—0.98	0.99 −0.21 to 0.22 0.0000 * −1.51 to −1.03
Q-DASH (scale 0–55)	Baseline Final	51.7 (7.48) 3.94 (3.11)	B vs. F—0.0001 *	52.1 (6.86) 51.6 (6.69)	B vs. F—0.93	0.99 −3.21 to 2.55 0.0000 * −49.7 to −45.6
S-LANSS (scale 0–24)	Baseline Final	14.1 (1.87) 3.24 (2.69)	B vs. F—0.0001 *	13.8 (1.87) 13.7 (1.71)	B vs. F—0.92	0.95 −0.52 to 0.97 0.0001 * −11.4 to −9.57

NOTE. Values are mean ± SD (range) or as otherwise indicated. Abbreviation: NT—neurodynamic techniques; ST—sham therapy; 2PD—two-point discrimination sense; CSPT—cutaneous sensory perception threshold; Q-DASH—Quick Disabilities of Arm Shoulder and Hand; S-LANSS—Leeds Assessment of Neuropathic Symptoms and Signs; n—number of participants; *p*—significance level, SD—standard deviation, CI—confidence interval, B—baseline, F—final. * Statistically significant difference.

## Data Availability

The data presented in this study are available upon reasonable request from the corresponding author due to privacy reasons.
